# Dopaminergic Degeneration and Small Vessel Disease in Patients with Normal Pressure Hydrocephalus Who Underwent Shunt Surgery

**DOI:** 10.3390/jcm9041084

**Published:** 2020-04-11

**Authors:** Tze-Wei Chang, Pao-Hui Tseng, Yi-Cheng Wang, Guo-Fang Tseng, Tsung-Lang Chiu, Shinn-Zong Lin, Sheng-Tzung Tsai

**Affiliations:** 1 Department of Neurosurgery, Hualien Tzu Chi Hospital/ Tzu Chi University, Hualien 970, Taiwan; kenny702187@gmail.com (T.-W.C.); bonne@tzuchi.com.tw (P.-H.T.); 520maitake@gmail.com (Y.-C.W.); poluschiou@gmail.com (T.-L.C.); shinnzong@yahoo.com.tw (S.-Z.L.); 2 Institiute of Medical Sciences, Tzu Chi University, Hualien 970, Taiwan; 3 Department of Anatomy, College of Medicine, Tzu Chi University, Hualien 970, Taiwan; guofang@mail.tcu.edu.tw

**Keywords:** normal pressure hydrocephalus, dopaminergic degeneration, small vessel disease, shunt, outcome

## Abstract

The diagnosis of idiopathic normal pressure hydrocephalus (iNPH) and the outcome of lumboperitoneal shunt treatment remains to be systematically explored. Here, we aim to evaluate whether the severity of dopaminergic degeneration and white matter small vessel disease could be predictors of outcome for iNPH patients subjected to lumboperitoneal shunt treatment. This is a single center retrospective study with 39 patients with probable iNPH undergoing programmable surgical lumboperitoneal shunt from June 2016 to March 2018 at Hualien Tzu Chi Hospital. In all patients, dopaminergic degeneration was determined with ^99m^Tc- TRODAT-1 SPECT scan, while white matter small vessel disease (Fazekas scale) was assessed with Brain MRI. The iNPH grading scale (iNPHGS) score and Karnofsky Performance Score (KPS) pre- and post-operation (6-month follow-up) were available for all patients. Linear regression was used to correlate the severities of dopaminergic degeneration and small vessel disease with lumboperitoneal shunt treatment outcomes. Their iNPHGS score improved significantly after surgery (pre-operatively, 7.8 ± 2.6; post-operatively, 5.7 ± 2.6 (26.9% improvement) (*p* < 0.05)). Moreover, the KPS was also improved significantly after surgery, by a mean of 24.6% from the baseline score (*p* < 0.05). A significant correlation was observed between the severity of dopaminergic degeneration and a poorer improvement of iNPHGS score (*p* = 0.03). However, improvement of the iNPHGS score was not correlated with white matter small vessel disease. Dopaminergic degeneration comorbidity neutralized the degree of improvement after surgery. Although white matter small vessel disease was correlated with iNPH incidence, it may not be a prognostic factor for shunt operation. These findings have implications for the use of dopaminergic imaging, as they might help predict the surgical outcome of patients with iNPH, while vascular mechanisms seem to be involved in iNPH pathophysiology.

## 1. Introduction

Idiopathic normal pressure hydrocephalus (iNPH) is an increasingly recognized disease among the elderly population that is associated with the clinical triad of gait disturbance, rapid cognitive impairment, and urinary incontinence [1,2]. iNPH involves the abnormal accumulation of cerebrospinal fluid (CSF) in the ventricular system of the brain. However, it remains a diagnosis without definitive and objective clinical signs, radiological findings, and pathological evidence, thus, further research is warranted to identify better predictors of the outcomes of this disease [3]. The difficulty in understanding the specific pathophysiology of iNPH exacerbates the problems associated with the definitive diagnosis of the condition and the referral of patients who would benefit the most from shunt surgery.

Many studies have suggested that vascular factors are strongly correlated with the development of iNPH and the various ensuing clinical characteristics [4]. Small vessel disease is identified by white matter hyperintensities on magnetic resonance imaging (MRI). It is a cerebrovascular disease that involves cerebral microvessels, and is strongly correlated with vascular factors [5]. In addition, many of the clinical manifestations of small vessel disease are similar to the symptoms of iNPH, such as cognitive decline, gait disability, and extrapyramidal symptoms; this depends on the underlying pathophysiology [5]. However, the manner in which the presence of small vessel disease might affect the surgical outcomes in patients with probable iNPH has not been addressed.

The differentiation between Parkinsonism, which is initially characterized by rest tremor, akinesia, and rigidity, and iNPH also rests on clinical acumen and neuroimaging, which may help elucidate the mechanism underlying the heterogeneous manifestations of dopaminergic neuron deficiency [6]. After adjusting for the neuroimaging of small vessel disease and comorbidities, studies have used dopaminergic imaging to identify typical patients with iNPH based on the diagnosis of iNPH mimics or parkinsonism [6,7]. Shunt surgery may ameliorate behavioral and cognitive deficits, and increase the level of striatal uptake of D2 receptors on imaging in patients with iNPH exhibiting reduced striatal postsynaptic uptake and the degeneration of dopaminergic neurons. Currently, studies that have correlated dopaminergic neuroimaging with clinical improvement after shunt surgery are limited.

The standard treatment for iNPH usually relies on shunt surgery, and ~50% patients exhibit a significant improvement after this intervention. Patients with clinical symptoms and radiological evidence of iNPH face deterioration in motor and cognitive symptoms and higher mortality if the condition is left untreated [8]. In addition, delaying the shunt surgery might preclude the full reversibility of the clinical signs [9]. As the etiology of iNPH and its related pathophysiology vary widely and are not fully known, the treatment outcomes might also vary and require evaluation, for several reasons. First, it is difficult to differentiate iNPH from neurodegenerative diseases, such as dementia or parkinsonism, at the early stage, even based on objective imaging findings [10]. Second, aged populations with iNPH usually have several medical comorbidities and cerebrovascular disease [11], which leads to a higher incidence of white matter small vessel disease [5]. Third, it has been estimated that <60% of patients with iNPH exhibit the full symptom triad, and that each symptom per se is not specific for the diagnosis of iNPH, as it is usually shared with other neurodegenerative diagnoses [12]. These data suggest that multi-modality neuroimaging biomarkers may be needed for the identification and differentiation of iNPH at an early stage, for a better prediction of surgical outcomes.

Our study aimed to evaluate how the presence of dopaminergic degeneration and small vessel disease might neutralize the improved clinical outcome of iNPH observed after shunt surgery.

## 2. Methods

### 2.1. Patient Selection and Clinical Evaluation

Physicians with expertise in hydrocephalus visited all referred patients with iNPH, who met the international guidelines for the clinical criteria of iNPH [13]. The following inclusion criteria were adopted: (1) age between 60 and 90 years; (2) presence of at least two of classical three symptoms; (3) ventriculomegaly with an Evans’ index >0.3, and a disproportionate high-convexity and medial subarachnoid space tightness on coronal MR images [14]; (4) absence of neurological disorders that combine with ventriculomegaly; and (5) normal intracranial pressure (lumbar tap initial pressure below 15 mmHg), with a positive response to a lumbar drainage test. All patients who exhibited severe lumbar spinal canal stenosis or vertebral scoliosis on plain radiograph or MRI were excluded from the study.

Before surgery, each patient underwent a clinical assessment that included scoring on the iNPH grading scale (iNPHGS), and Karnofsky Performance Score (KPS) [15]. Pre-operative brain MRI was obtained to evaluate structural abnormalities and small vessel disease. Ethical approval was obtained from the institutional review board of Tzu Chi General Hospital. We conducted follow-up visits for all patients 6 months after the shunt surgery.

### 2.2. Imaging

#### 2.2.1. MRI

The pre-operative MRI was performed on a 1.5-tesla MR unit (SIGNA HDXT 1.5T General Electric healthcare, Milwaukee, WI, USA). The standard protocol consisted of the acquisition of T1-weighted axial images at a thickness of 0.75 mm, T2-weighted axial images at a thickness of 2 mm (echo time [TE], 100–120 ms; repetition time [TR], 4000–6000 ms; voxel size, 1 × 1 × 5 to 7.5 mm^3^; 19 to 24 slices), axial fluid-attenuated inversion recovery (FLAIR) images (TE, 100–140 ms; TR, 6000–10,000 ms; inversion time, 2000–2400 ms; voxel size, 1 × 1 × 5 to 7.5 mm^3^; 19 to 24 slices), and coronal 3D T1 sequence (TE, 4–7 ms; TR, 10–25 ms; flip angle, 15°–30°; voxel size, 1 × 1 × 5 to 1.5 mm^3^). Each of these sequences was performed on contiguous slices pre-operatively.

**Figure 1:**
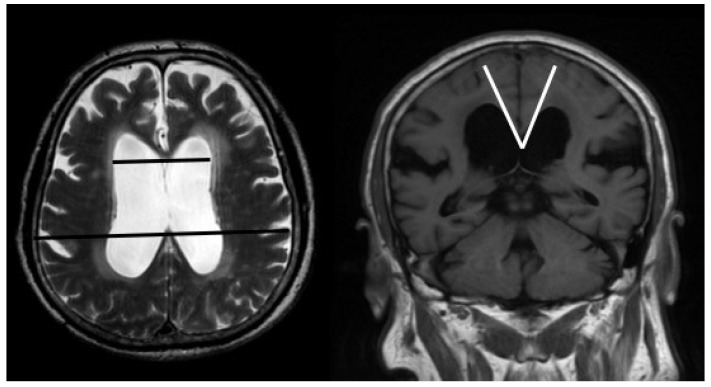
Pre-operative brain MRI (magnetic resonance imaging) of one representative patient with iNPH (idiopathic normal pressure hydrocephalus), who showed an increased Evans’ index (>0.3) and a sharp callosal angle (<90°).

Measurements of ventricular enlargement (Evans’ index >0.3) and callosal angle were performed pre-operatively by an independent rater (Figure 1). According to previous studies, callosal angle should be measured between the lateral ventricles on a coronal MR image through the posterior commissure, perpendicular to the anterior commissure–posterior commissure plane [14,16]. In order to obtain a coronal image, multiplanar reconstruction was done for each patient. In cases in which such thin images had not been acquired, the measurements were performed on an interactively reconstructed coronal image from a sagittal scan in a plane that is perpendicular to the anterior commissure–posterior commissure plane.

Regarding the staging of small vessel disease and white matter hyperintensity, we rated age-related white matter changes based on four scales of Fazekas scores (range, 0–3) from the axial FLAIR images with a slice thickness of 5 mm [17,18]. These images were examined by experienced and independent radiologists. We defined periventricular white matter hyperintensities as lesions that were larger than 5 mm on FLAIR images. We evaluated changes in the basal ganglia in the same way and considered white matter lesions even if they were located in the gray matter nuclei, which contain a small amount of white matter.

#### 2.2.2. 99 ^m^Tc-TRODAT-1 SPECT

**Figure 2:**
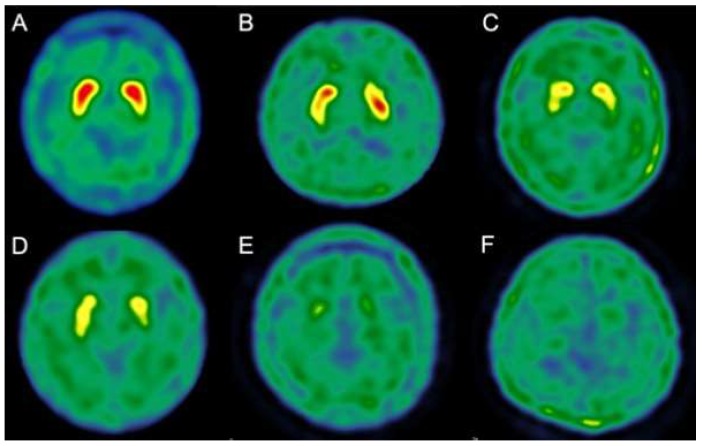
Different staging of ^99 m^Tc-TRODAT-1 SPECT for patients with iNPH. A. Normal. B. Stage 1. C. Stage 2. D. Stage 3. E. Stage 4. F. Stage 5. A higher stage implies a greater loss of dopaminergic neurons.

^99 m^Tc-TRODAT-1 was prepared as described previously [19]. In summary, patients were placed in a supine position with their head fixed. Each patient received a single bolus injection of 740 MBq ^99 m^Tc-TRODAT-1 and 15 dynamic images of the brain were acquired over 30 min. Images were reconstructed using back-projection with a Metz filter. Regions of interest were marked on one side of the striatum, as reference for the corresponding MR images, and were fitted to the contralateral side. We marked regions of interest over the whole striatum, including bilateral caudate nucleus and putamen on composite images of the three slices with the highest TRODAT-1 basal ganglia activity [20]. We classified these findings in relation to the uptake of ^99 m^Tc-TRODAT-1. The details of the staging procedure were as described previously [19]. Briefly, we classified these findings in relation to the uptake of ^99 m^Tc-TRODAT-1 as follows: Stage 1, diminished uptake over the unilateral putamen; Stage 2, diminished uptake over the bilateral putamen; Stage 3, diminished uptake over the unilateral caudate and bilateral putamen; Stage 4, diminished uptake over the bilateral caudate and bilateral putamen; Stage 5, negligible uptake over the bilateral caudate and bilateral putamen (Figure 2).

### 2.3. Surgical Procedures

The patients included in this study were administered a lumbar drainage test over 3 days, to identify subjective or objective responders. All responders underwent implantation of a lumboperitoneal shunt with a programmable pressure valve (programmable Strata NSC valve and SL 43555; Medtronic Neurosurgery, Medtronic Inc., Dublin, Ireland) under general anesthesia. Using a paramedian approach, a spinal catheter with a length of 15 cm was inserted through the L3/4 or L4/5 interlaminar space into the subarachnoid space. After the creation of a small subcostal pouch over the abdominal side, a subcutaneous tunnel was made, and the ventral tip of the spinal catheter was passed to the abdominal side. The spinal catheter was connected to the reservoir of the valve. The abdominal catheter was trimmed to 50 cm and inserted into the abdominal cavity after spontaneous CSF drainage was confirmed.

The initial pressure was set to a level of 2.0. If the patient’s symptoms did not improve after shunt implantation (at the 1-month follow-up), we lowered the pressure setting to one level (0.5) below the initial one. After adjusting the valve pressure, we set the pressure to one level lower if the extent of the benefit from surgery was not improving continuously or did not reach the status before the development of symptoms. However, in cases of development of overdrainage, we followed the patients closely to determine if an increase in the pressure setting was necessary immediately.

### 2.4. Statistical Analyses

We used the Wilcoxon signed-rank test to compare the differences between pre-operative and post-operative clinical outcomes (including clinical scoring and pressure gradient during lumbar puncture). Correlations between staging of ^99 m^Tc-TRODAT-1 and small vessel disease scoring were obtained using the Spearman rank correlation (*R*^2^). Significance was set at *p* < 0.05. There were no missing data and outliers were included in the analyses.

## 3. Results

From June 2016 to March 2018, we reviewed retrospectively consecutive patients with iNPH who underwent shunt surgery (*n* = 39). The mean age was 75 years and 66.67% (*n* = 26) of the patients were men. Detailed demographics and clinical characteristics, including comorbidities, are described in Table 1. The 39 patients fulfilled the criteria of probable iNPH. History of hypertension accounted for 58.97 % and type II diabetes mellitus accounted for 23.08% of the cohort. Their mean Evans’ index was 0.31 ± 0.05 and the callosal angle was 92.7° ± 22.8°. Pre-operatively, their mean iNPHGS score was 7.8 ± 2.6. After objective or subjective symptomatic improvement in the lumbar drainage test, these patients with iNPH underwent lumboperitoneal shunt surgery. At a mean follow-up time of 6 months, their iNPHGS score improved significantly to 5.7 ± 2.6 (26.9% improvement) (*p* < 0.05) (Table 2). The KPS were also significantly improved after shunting (*p* < 0.05).

### 3.1. Association between Clinical Characteristics and Imaging Biomarkers of ^99 m^Tc-TRODAT-1 SPECT and White Matter Small Vessel Disease

The mean pre-operative staging from ^99 m^Tc-TRODAT-1 imaging was 2.1 ± 1.2. There were 19 patients at stage 0, followed by 5 patients at stage 1, 5 patients at stage 2, 7 patients at stage 3, 2 patients at stage 4, and 1 patient at stage 5. The degrees of dopaminergic degeneration on TRODAT-SPECT imaging were inversely correlated with the improvement in total iNPHGS score (*p* = 0.03, *R*^2^ = –0.62). The analysis of the different symptom domains of iNPH revealed that the severity of the degeneration of dopamine on imaging was only significantly correlated with the cognitive improvement observed from the pre-operative to post-operative status (*p* = 0.02, *R*^2^ = –0.28), and not with gait or urinary symptoms (*p* = 0.13 and *p* = 0.11, respectively). The stages of ^99 m^Tc-TRODAT-1 were also significantly correlated with the improvement of KPS observed 6 months after surgery (*p* < 0.05). In contrast, the visually rated Fazekas scores were not correlated with the improvement of iNPHGS score (*p* > 0.05). Finally, the severity of small vessel disease was not correlated with cognitive, gait, or urinary symptoms (*p* > 0.05).

### 3.2. Adverse Effects

Among the 39 patients who underwent lumboperitoneal shunt surgery, two serious adverse effects related directly to the surgery were reported. One patient developed chronic subdural hematoma requiring drainage surgery about 2 months after shunt placement. His hemiparesis recovered fully after hematoma drainage. Another patient’s subdural hematoma recovered after we adjusted the pressure setting from level 1.0 to 2.0. In addition, there were three cases of non-serious adverse effects, including positional headache, intermittent neck stiffness and asymptomatic subdural fluid collection. All of these complaints resolved after increasing the pressure setting of the shunt reservoir. One patient exhibited recurrence of the iNPH symptoms, and shunt tube (intrathecal tube) migration was detected about 2 months after surgery. The patient’s symptoms improved gradually after we repositioned the lumbar end of the tube.

## 4. Discussion

Our cohort study showed that concomitant dopaminergic neurodegeneration and small vessel disease were not uncommon among patients with probable iNPH. These comorbidities also exhibited effects of a varying degree on the severity of iNPH, and on the response to its treatment. In accordance with previous reports, we found that a history of hypertension and DM was much more common among patients with iNPH, and detected a strong association between the incidence of small vessel disease and iNPH, which suggested that a better control of vascular risk factors might lead to a lower incidence of iNPH, or a better outcome of CSF drainage intervention [11,21]. However, the coexistent small vessel disease did not predict the outcomes of shunt surgery for iNPH in our study. In addition, a linear regression analysis revealed that dopaminergic degeneration was an independent risk factor for iNPHGS improvement after shunt intervention. We demonstrated that dopaminergic neurodegeneration, as assessed using ^99 m^Tc-TRODAT-1 SPECT, might decrease the benefit of symptomatic improvement observed after lumboperitoneal shunt surgery for iNPH.

Atherosclerotic diseases, including coronary artery disease, essential hypertension, and peripheral arterial disease, occur with increased frequency among patients with iNPH compared with controls [11]. These vascular factors and ensuing worse small vessel disease contribute to the increased incidence of iNPH [21]. A volumetric analysis of small vessel disease revealed a correlation with motor and cognitive symptoms [22]. Patients with iNPH also have a significantly higher prevalence and worse severity of periventricular white matter disease on MRI [23]. These associations have led researchers to hypothesize that chronic ischemia around the periventricular area leads to increased compliance of the ventricular wall and gradual ventricular enlargement, because of normal fluctuations of intracranial pressure. Intraventricular pulse pressure and systemic hypertension have also been correlated with hydrocephalus. Pioneering studies by Eide et al. further showed monitored ICP and glymphatic MRI may not only delineate the mechanisms but identify surgical response for iNPH patients [24,25]. In addition, periventricular ischemia may result in locally increased venous resistance, decreased CSF absorption, and ventricular enlargement. Boon et al. have shown a good correlation between decreased CSF absorption and outcomes of shunt surgery [26]. Arachnoid thickening is a common autopsy finding in patients with iNPH, as it is present in up to 50% of cases [27]. Ultrasound-based study of 20 patients with iNPH reported that findings of retrograde flow in the internal jugular veins during the Valsalva maneuver were more likely to occur in patients compared with controls (95% vs. 25%) [28]. In addition, brain MRI imaging identified the correlation between venous hypertension and the development of chronic hydrocephalus [29,30,31]. These studies support the contention that elevated central venous pressure and impaired CSF absorption lead to a higher incidence of iNPH. Therefore, acetazolamide, which is a drug that is used commonly as a diuretic and to lower blood pressure, not only reverses small vessel disease on MRI imaging, but also strongly predicts a better symptomatic amelioration of iNPH [23,32]. This finding is similar to those obtained for CSF diversion by surgical shunting [33].

Although parkinsonism is estimated to be present in ~20% of iNPH cases, this association does not seem to correlate with the poorer improvement observed after shunt surgery, and is independent of concomitant white matter changes [34]. The levodopa challenge test has been useful to identify patients with Parkinson’s disease who were initially diagnosed as having iNPH [35]. In addition, Allali et al. used dopaminergic imaging ([123I]FP-CIT SPECT) to differentiate iNPH from iNPH mimics (iNPH plus parkinsonism) [6]. In vivo studies also have found the causal relationship of change in cerebral monoamine neurotransmitters after induced animal models of hydrocephalus [36,37]. Our study identified a linear correlation between dopaminergic degeneration and the improvement of iNPHGS scores after shunt surgery, which further indicates that iNPH is a neurodegenerative disease that might involve the dopaminergic neuronal system. Therefore, the establishment of functional neuroimaging markers, such as dopaminergic imaging, might facilitate the identification of better iNPH candidates for referral to shunt surgery and the prediction of surgical efficacy.

As the main pathophysiology of patients’ symptoms is derived from excessive accumulation of CSF in cerebral ventricular systems, clinical improvement of iNPH can be achieved mainly by shunt implantation surgery aimed at diverting the flow and absorption of CSF. This results in ameliorations of up to 50% and 80% in the symptoms of NPH [38]. To select candidates for shunt surgery, prognostic information is gathered from clinical examination, neuroimaging, and CSF testing [3]. Currently, the radiological imaging findings that suggest a clinical suspicion of NPH include dilated ventricles, effaced sulci at the high convexities, a sharp callosal angle over the lateral ventricles, and signs of hyperdynamic CSF flow [14]. These radiological signs are considered as being useful to differentiate NPH from other coexisting neurodegenerative diseases; however, their roles in the prediction of the responsiveness to shunt surgery have not been established [39].

Although shunt implantation (either ventriculoperitoneal or lumboperitoneal shunt) ameliorates the motor and cognitive (partly) disabilities of NPH, the extent to which surgery mitigates individual symptoms still varies widely among these patients. Currently, physicians rely mostly on the different clinical features (symptoms) of patients for iNPH diagnosis and prediction of treatment responsiveness [40]. Brain scans may help the diagnosis of this disease and are usually employed to detect secondary structural lesions, such as neurodegenerative diseases, stroke, or traumatic brain injury, which exhibit a similar and ambiguous disease presentation [10]. Even if the lumbar tap test or lumbar drainage test provides a better predictive value for the diagnosis of iNPH, studies aimed at identifying clinical, radiological, and neurophysiological biomarkers of this disease may facilitate a proper diagnosis and promote the effectiveness of treatment with shunt surgery [41]. With regard to the surgical options, one comparative study between lumboperitoneal shunt with programmable valves and ventriculoperitoneal shunt showed similar efficacy and safety [42]. Studies including meta-analysis and systemic review also demonstrated that the benefit and risks between various CSF diversion strategies, including lumboperitoneal shunt, ventriculoperitoneal shunt and endoscopic third ventriculostomy are similar [43,44]. In our study, we found that the benefit and risk of using lumboperitoneal shunt for iNPH patients are also comparable with the literature. 

Some patients with iNPH are later diagnosed with comorbidities, such as parkinsonism or neurodegenerative dementia, or have evidence of “dual pathology” at the time of shunting [10,45,46]. For example, cerebral pathology with β-amyloid in patients undergoing shunt surgery for iNPH were linked with a poorer improvement after operation [47,48]. Such cases have suggested that neurodegeneration, such as Alzheimer’s disease, may play a role in ventricular enlargement and iNPH. The suboptimal long-term outcomes for these initially diagnosed iNPH cases, and the association with another neurodegenerative disease, suggest that factors that can identify these cases before shunt surgery are a prerequisite for clinicians to weigh the risks and benefits of this intervention. Another recent study showed that patients with iNPH have low CSF β-amyloid 42 and t-tau and p-tau levels [49]. In addition, the levels of these proteins were lower in the cerebral ventricles compared with the lumbar CSF. These reports suggest that the observed reductions in β-amyloid peptide, soluble β-amyloid precursor protein fragment, and tau protein levels could represent a decrease in the clearance from the extracellular fluid, because of reduced centripetal flow of the extracellular fluid caused by the retrograde CSF dynamics of iNPH [50]. This also highlights the possibility of using CSF biomarkers to predict the effectiveness of shunt surgery in patients with iNPH. Furthermore, another brain biopsy study in iNPH patients also identified the possible role of aquaporin-4 water channels and associated disrupted blood-brain barrier in the pathophysiology of iNPH, which implies that novel therapy for iNPH might be feasible through restoring this impaired aquaporin distribution and integrity of blood-brain barrier [51,52,53]. 

Our preliminary study has several limitations. First, it showed significant correlation between outcomes of iNPH patients undergoing shunt surgery and degrees of dopaminergic degeneration revealed on SPECT imaging. However, we did not perform correlation analyses between clinical outcomes (iNPHGS and KPS) and striatal dopaminergic vs Fazekas scale. Second, this relatively small cohort limits the statistical power to discern small differences in clinical outcomes and brain imaging (SPECT and MRI). Third, ^99 m^Tc-TRODAT-1 SPECT of three patients were at stage 4~5. Although they had the least improvement after surgery, they had temporary improvement during the lumbar drain trial. it is very debatable to consider these patients to have iNPH. They would most probably have a degenerative parkinsonism, which would preclude a diagnosis of iNPH. This further supports the feasibility and benefit of dopaminergic imaging before including probable iNPH patients for shunt surgery. Finally, as we mentioned, a sharp callosal angle has been considered as a strong positive predictor for a better surgical outcome. From our knowledge, the preoperative callosal angel has not been defined as one of absolute criteria for iNPH diagnosis. Therefore, we included patients mainly based on the response of lumbar drainage test. This may explain why some patients had callosal angles larger than 90 degrees. 

## 5. Conclusions

iNPH is usually associated with the elder population, and with several vascular comorbidities and neurodegenerative diseases. The correlation between dopaminergic degeneration and the surgical outcome of shunt surgery for iNPH has important implications, as early effective treatment of iNPH relies on the consideration of underlying heterogeneous pathophysiological presentations. A larger and longer cohort-based study is warranted to incorporate multi-modality diagnostic and therapeutic tools and identify specific biomarkers of iNPH, including clinical scales, radiological imaging, and protein profiles in the CSF.

## Figures and Tables

**Table 1:** Demographics of normal pressure hydrocephalus patients (*n* = 39).

Age (Mean ± SD)	75 ± 9.9
Male, Sex	26 (66.67%)
HTN	23 (58.97%)
DM	9 (23.08%)
CAD	3 (7.69%)
CKD	6 (15.38%)
BPH	10 (38.46%)

HTN (hypertension); DM (Diabetes Mellitus); CAD (coronary artery disease); CKD (Chronic kidney disease); BPH (Benign Prostatic Hyperplasia).

**Table 2:** Clinical staging of imagings, Karnofsky Performance Scale, iNPHGS and intra-cranial pressure gradient of normal pressure hydrocephalus patients (*n* = 39).

**^99 m^Tc-TRODAT-1 stages**	
Average	2.1 ± 1.2 (0~4)
**White matter small vessel disease (Fazekas scores)**	
Periventricular area	1.6 ± 0.9 (0~3)
Putamen	0.4 ± 0.5 (0~2)
**Karnofsky Performance Scale**	
Pre-operative	56.9 ± 11.8 (30~80)
Post-operative	71 ± 11.9 (50~90)
**iNPHGS**	
Pre-operative scores	7.8 ± 2.6 (5~12)
Cognition	2.3 ± 1 (1~4)
Gait	2.9 ± 0.8 (2~4)
Urinary function	2.5 ± 1.3 (1~4)
Post-operative scores	5.7 ± 2.6 (5~9)
Cognition	2 ± 1 (1~3)
Gait	1.7 ± 0.8 (0~2)
Urinary function	2.0 ± 1.3 (0~3)
**Intra-cranial pressure (lumbar puncture)**	
Opening pressure	11.7 ± 4.6 (4~20)
Close pressure	5.4 ± 3.1 (0~13)
Pressure gradient	6.5 ± 2.9 (3~14)

iNPHGS: idiopathic normal pressure hydrocephalus grading scale.
